# Improved inference of tandem domain duplications

**DOI:** 10.1093/bioinformatics/btab329

**Published:** 2021-07-12

**Authors:** Chaitanya Aluru, Mona Singh

**Affiliations:** Department of Computer Science and Lewis-Sigler Institute for Integrative Genomics, Princeton University, Princeton, NJ 08540, USA; Department of Computer Science and Lewis-Sigler Institute for Integrative Genomics, Princeton University, Princeton, NJ 08540, USA

## Abstract

**Motivation:**

Protein domain duplications are a major contributor to the functional diversification of protein families. These duplications can occur one at a time through single domain duplications, or as tandem duplications where several consecutive domains are duplicated together as part of a single evolutionary event. Existing methods for inferring domain-level evolutionary events are based on reconciling domain trees with gene trees. While some formulations consider multiple domain duplications, they do not explicitly model tandem duplications; this leads to inaccurate inference of which domains duplicated together over the course of evolution.

**Results:**

Here, we introduce a reconciliation-based framework that considers the relative positions of domains within extant sequences. We use this information to uncover tandem domain duplications within the evolutionary history of these genes. We devise an integer linear programming approach that solves our problem exactly, and a heuristic approach that works well in practice. We perform extensive simulation studies to demonstrate that our approaches can accurately uncover single and tandem domain duplications, and additionally test our approach on a well-studied orthogroup where lineage-specific domain expansions exhibit varying and complex domain duplication patterns.

**Availability and implementation:**

Code is available on github at https://github.com/Singh-Lab/TandemDuplications.

**Supplementary information:**

Supplementary data are available at *Bioinformatics* online.

## 1 Introduction

Nearly all proteins contain short, modular subsequences known as domains. These domains have structural and functional properties, and can be categorized into domain families based on sequence similarity. The number of domains present in a protein varies widely, ranging from a single domain to over a hundred in some protein families (Labeit *et al.*, 1990). Differing combinations of domains occur across multidomain protein sequences, and changes in the repertoire and number of domains are a major driver of protein evolution (Chothia *et al.*, 2003). Understanding the domain-level evolutionary processes behind these changes is crucial to understanding the evolution of sequence families in general.

Proteins typically diversify their repertoire of domains through domain duplications and losses. Duplications may be either single domain duplications or tandem duplications, in which several consecutive domains within a protein duplicate at once in a single event (Björklund *et al.*, 2006). For proteins with multiple consecutive repeats of the same domain, it can be difficult to ascertain which types of duplications gave rise to those repeats. However, uncovering the evolutionary history of protein domains can be instructive in learning their function. For example, nebulin domains have been observed to duplicate in sets of seven (Björklund *et al.*, 2010). These match the seven-actin monomer repeat unit of the actin filament to which they bind during muscle contractions (Labeit *et al.*, 2011). In other cases, the same protein family may exhibit several modes of duplication. For example, filamin domains in the Filamin-A protein family have been shown to exhibit both tandem duplications and consecutive single duplications (Light *et al.*, 2012). Proteins with repeated copies of the same domain are ubiquitous across the tree of life, and account for nearly 20% of human proteins (Björklund *et al.*, 2006).

Evolutionary event histories are typically gleaned through the process of reconciliation (Goodman *et al.*, 1979; Li and Bansal, 2019; Muhammad *et al.*, 2018). In these methods, a phylogeny of related domains is built, along with a phylogeny of the genes (and sometimes species; Bansal *et al.*, 2012; Stolzer *et al.*, 2015) from which they came. Nodes in the domain phylogeny are then mapped to nodes in the gene phylogeny according to a set of biological constraints. This mapping is used to infer domain duplications and losses (and sometimes other events, including transfers, merges and splits; Hallett *et al.*, 2004; Yi-Chieh *et al.*, 2012). Duplications are typically handled in one of two ways. In the traditional approach, multidomain duplications are ignored. Instead duplications are assumed to occur one domain at a time. In other approaches, multidomain duplications are explicitly included in the model (Aluru and Singh, 2020; Bansal and Eulenstein, 2008; Dondi *et al.*, 2019; Guigo *et al.*, 1996). However, these approaches do not consider tandem duplications. Instead, they try to minimize the total number of multiple duplication events without constraining the positions of the domains involved. This can lead to groupings of domains that are scattered across the sequence and could not have duplicated together in a single event. In practice, these types of models are acceptable for simple cases with few duplication events, but quickly lose accuracy for proteins with repeated domain duplications, where cascades of independent duplication events can be incorrectly grouped into a few large duplication groups.

Our main contribution is a reconciliation framework, which significantly increases the accuracy of tandem duplication inference. We (i) introduce a model for tandem duplication events, (ii) integrate this model into the reconciliation framework, (iii) give both an exact integer linear programming (ILP) solution and a significantly faster heuristic for the problem and (iv) show that we can accurately identify tandem duplications in both simulated and real data. We prove that our framework can correctly identify tandem duplications in the absence of domain shuffling and losses, and show in simulation that our methods are robust even under high loss scenarios.

The rest of the article is organized as follows. Section 2 gives definitions useful for understanding our framework and defines the Tandem Duplication Loss (TDL) reconciliation problem. Section 3 gives our tandem duplication model and shows how to use the position of domains within modern day sequences to differentiate single and tandem duplications. Section 4 introduces our heuristic solution, while Section 5 demonstrates the performance of our methods on both real and simulated datasets.

## 2 The TDL reconciliation problem

In this section, we give a description of the TDL reconciliation problem. We first introduce notation which will be useful in describing the problem, and then present our reconciliation formulation for finding multidomain duplications. We define tandem duplications, add constraints to distinguish between tandem and single duplications, and finally present a parsimony framework by which to optimize reconciliations. While we describe the reconciliation problem in terms of domain and gene trees, we note that it is equally applicable in the context of reconciling gene and species trees.

### 2.1 Preliminaries

Domain-gene reconciliation requires as input a rooted full binary gene tree and a rooted full binary domain tree. We refer to these phylogenies as the gene tree *G* and the domain tree *D*, respectively. For any rooted full binary tree *T*, we denote the sets of vertices, internal vertices, leaves and edges by V(T),I(T),L(T) and *E*(*T*), respectively. A directed edge, or arc, in *T* between parent node *u* and child *v* is denoted as (*u*, *v*). The parent of node *v* in tree *T* is denoted as $p_{T}(v)$. If there is a path from *u* to *v* in tree *T*, we say that *v* is a descendant of *u* in the tree *T* (or equivalently that *u* is an ancestor of *v* in *T*), and we denote this by $v\leq_{T}u$ and by $v{<}_{T}u$ if v≠u (or equivalently by $u\geq_{T}v$ and by $u{>}_{T}v$ if v≠u). Vertices *u* and *v* are incomparable in *T* if $u{≰}_{T}v$ and $v{≰}_{T}u$, and otherwise are comparable in *T*. We define the lowest common ancestor (LCA) of nodes *x* and *y* in tree T, termed ${\text{LCA}}_{T}(x,y)$, to be the vertex *v* in *T* such that $v\geq_{T}x$ and $v\geq_{T}y$, and for which there is no other vertex *u* where $u{<}_{T}v$ and $u\geq_{T}x$ and $u\geq_{T}y$. For ease of notation, we omit the subscript *T* when it is obvious. The distance between comparable vertices *u* and *v* in *T*, ${\text{dist}}_{T}(u,v)$, is defined as the number of arcs in the path from *u* to *v* if u≥v and otherwise in the path between *v* and *u* in *T*. We denote the subtree of tree *T* rooted at internal node *u* as *T_u_*.

A leaf mapping σ:L(D)→L(G) maps each leaf node in the domain tree to a leaf in the gene tree. A full mapping γ:V(D)→V(G) maps each node in the domain tree to a node in the gene tree. Each gene may contain multiple domains, but every domain instance occurs in exactly one gene. Therefore, these mappings are many-to-one. A full mapping must be consistent with a leaf mapping (i.e. σ(u)=γ(u) for all leaves *u* in the domain tree).

### 2.2 TDL reconciliations

**Fig. 1.**
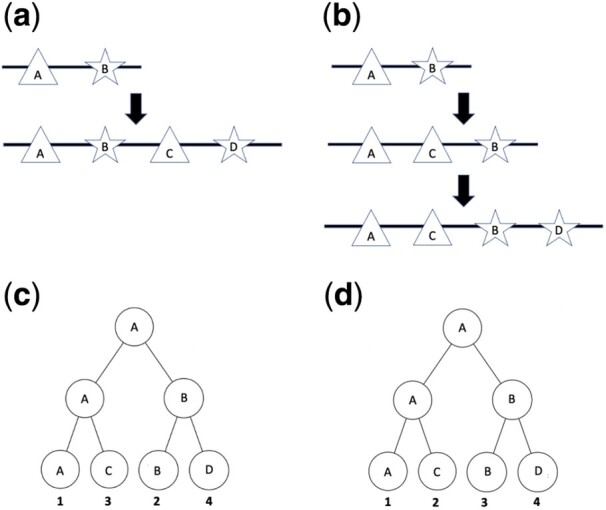
Two possible duplication patterns and their corresponding phylogenetic trees. (**a**) A protein has two domain instances, depicted by a triangle and star, labeled ‘A’ and ‘B’, respectively. Here, a single tandem duplication occurs, and the entire stretch containing both domains is duplicated at once. The resulting protein contains two instances each of the star and triangle domains. Without loss of generality, after duplication we refer to the first copy of the two domains in the second sequence as corresponding to the ancestral domains and thus refer to them by ‘A’ and ‘B’. The domains in the second copy are considered new domains and are referred to by the new labels ‘C’ and ‘D’. (**b**) A different set of duplication events with the same starting protein. Here, we see two individual domain duplications; first the triangle domain duplicates and then the star domain duplicates. In the end, the protein has two triangle and two star domains, as in part (a). Again, after duplication the first copy of each of these two domains is assumed to be the ancestral one. (**c** and **d**) Phylogenetic trees built from the domains found in the final proteins from parts (a) and (b). The leaf labels correspond to the name of the domain, with position in the sequence marked below. Ancestral node labels are obtained by taking the name of its leftmost child (as by design this is assumed to be the original copy after the duplication). Their positions are unknown and must be inferred. Note that the topologies of the trees are identical, so we cannot distinguish between tandem duplication and two individual duplication events from tree topology alone. However, by including the relative position of each domain in the tree, we see that the leaves in (c) are interleaved, indicating a tandem duplication, while the leaves in (d) are not, indicating that two separate duplication events occurred.

Given domain and gene trees, the goal of reconciliation is to assign an evolutionary event to each internal node in the domain tree and pinpoint the gene in which it existed. To determine which ancestral gene a given domain was present in, reconciliation methods infer *γ*, the full mapping from nodes in the domain tree to nodes in the gene tree. While inferring *γ*, a reconciliation also assigns an evolutionary event to each internal node in the domain tree. We consider three types of evolutionary events: co-duplications, tandem duplications and losses. Co-duplications are domain duplications that occur as a result of gene tree bifurcations, typically due to whole gene duplications or speciation events. Tandem duplications are duplications of one or more consecutive domains within a gene. To ensure that domains grouped together as taking part in the same duplication are actually part of a tandem duplication, we extend the typical reconciliation formulation to additionally use a pairwise eligibility matrix *E*. For any two nodes *u* and *v* in the domain tree, *E_uv_* = 1 if *u* and *v* could be part of the same tandem duplication, and 0 otherwise. Briefly, *E_uv_* is computed using the relative positions of the children of *u* and *v* on the gene. If their children are interleaved (see Fig. 1), then *u* and *v* are considered pairwise eligible, and otherwise not. For ancestral domains, whose positions on the sequence are unknown, we must infer relative orderings. In the next section, we describe how this inference is done, and how eligibility matrices are computed from extant sequences, domain trees and gene trees. Losses correspond to single domain losses within a gene; these do not label nodes within the domain tree as they are not observed, and will be inferred instead (as described further below). We do not consider other possible evolutionary events (e.g. transfer of domains between contemporary sequences).

More formally, given a gene tree *G*, a domain tree *D*, a leaf mapping σ:L(D)→L(G), and an eligibility matrix *E*, a TDL reconciliation consists of a full mapping γ:V(D)→V(G), a set Σ that consists of all nodes in *D* that are associated with co-duplications, and a set Δ where every tandem duplication is represented as the set of nodes in *D* corresponding to domains duplicated in that event. Note that within Δ, a duplication of a single domain *u* is represented as {*u*}, whereas a tandem duplication of domains $u_{1},u_{2},\ldots ,u_{m}$ is represented as $\left\{u_{1},u_{2},\ldots u_{m}\right\}$. Every node in *I*(*D*) is assigned either a co-duplication or a tandem duplication event. Every non-leaf domain node is assigned exactly one event, and therefore Σ and the sets contained in Δ together form a partition of *I*(*D*).

#### 2.2.1 Valid TDL reconciliations

A TDL reconciliation must maintain biological consistency with the input trees *G* and *D*, mapping *σ* and eligibility matrix *E*, and thus *γ*, Σ and Δ are constrained. A valid TDL reconciliation is defined as follows:

Definition 1 (TDL Reconciliation Problem). *Input: A full rooted binary gene tree G, a full rooted binary domain tree D, a leaf mapping* σ:L(D)→L(G)  *and a symmetric, binary* |V(D)|×|V(D)|  *eligibility matrix E. Output: A full mapping* γ:V(D)→V(G)*, a set of co-duplication nodes* Σ⊆I(D), *and a set of tandem duplication sets* $\Delta =\{X_{i}:X_{i}\subseteq I(D)\}$  *such that:*

1.
  -
γ(u)=σ(u) ∀  u∈L(D)
  - *For any* u,v∈V(D)  *s.t.* $p_{D}(v)=u,\;\gamma (v)\leq_{G}\gamma (u)$
2. *For any* u,v,w∈V(D)  *where* $p_{D}(v)=p_{D}(w)=u$,
  -
u∈Σ *if and only if* γ(v)  *and* γ(w)  *are incomparable in G*
  -
$u\notin \Sigma \Leftrightarrow u\in \cup_{X\in \Delta }X$

3. *For each* X∈Δ  * where* $X=\{x_{1},\ldots ,x_{m}\}$
  - *x_i_ and x_j_ are incomparable in D* $\forall x_{i},x_{j}\in X$
  -
$\gamma (x_{i})=\gamma (x_{j})\;\forall x_{i},x_{j}\in X$

  -
$E[x_{i}][x_{j}]=1$ $\forall x_{i},x_{j}\in X$
4. *For any* X,Y∈Δ  *where* $X=\{x_{1},\ldots ,x_{m}\}$  *and* $Y=\{y_{1},\ldots ,y_{n}\}$, $∄(x_{i},x_{j},y_{k},y_{l})$  *s.t.* $x_{i}{>}_{D}y_{k}$  *and* $x_{j}{<}_{D}y_{l}$

These conditions combine our understanding of domain evolution with constraints implied by the inputs. The first set of constraints deal with domain to gene node mapping. A full mapping must respect the input leaf mapping. Because we do not allow horizontal domain transfers in our model, domains must follow the evolutionary history of their encompassing genes. If a domain is mapped to a particular gene, then its children must be mapped to that gene or descendants of that gene. The second rule is used to decide which event a domain is assigned. A domain *d* present in gene *g* may only be considered a co-duplication if the children of *d* are present in incomparable descendants of *g*. Otherwise, *d* is assigned a tandem duplication event. Conditions 3 and 4 constrain tandem duplication sets. Condition 3 states that for any pair of domains to be in a tandem duplication set, they must (i) have no ancestry relationship between the two, (ii) have been present in the same gene and (iii) be allowed to be in the same tandem duplication set by the eligibility matrix. Finally, condition 4 ensures temporal consistency between tandem duplication sets.

These constraints only pertain to tandem duplication and co-duplication events. Lost domains are not seen in existing genes and therefore do not show up in the input domain tree. Losses are instead inferred based on the mapping of domains to genes. For each parent–child pair (u,v)∈E(D), we infer one loss for each gene tree vertex on the path between γ(u) and γ(v); in this case, at each such intermediary node in *G*, there are no descendants of domain *u* observed in the other branch of the gene tree. We note that if *u* is a co-duplication node, $\mathrm{dis}t_{G}(\gamma (u),\gamma (v))\geq 1$, as its domain duplication arises from a gene bifurcation represented in *G*. An additional loss is inferred if u∉Σ (i.e. *u* is part of a tandem duplication) and γ(u)≠γ(v); in this case, in addition to one loss for each intermediary node in *G*, there is another loss that must have existed at node γ(u) corresponding to a co-duplication that we do not see in the domain tree. Across all edges in the domain tree, the total number of inferred losses is

$$\left(\sum_{\left(u,v)\in E(D\right)}d{\text{ist}}_{G}(\gamma (u),\gamma (v))\right)-2|\Sigma |.$$

#### 2.2.2 Maximum parsimony costs for TDL reconciliations

For any input, there may be several valid reconciliations. We use a parsimony framework with event costs to choose the optimal one among them. Co-duplication events are required due to gene- or species-level events, and therefore have 0 cost. Tandem duplications are assigned a fixed cost *c_D_* per set (regardless of size), while losses are assigned a cost *c_L_*. The full cost of a TDL reconciliation is given by

$$C=c_{D}\times |\Delta |+c_{L}\times \left[\left(\sum_{\left(u,v)\in E(D\right)}d{\text{ist}}_{G}(\gamma (u),\gamma (v))\right)-2|\Sigma |\right].$$

#### 2.2.3 Relationship of the TDL reconciliation formulation to previous formulations

Reconciliation has typically been used to infer gene-level events, with a few recent works extending the framework to domains. In the earliest reconcilation models, known as Duplication Loss (DL) models, duplications are restricted to consist of single domains (or genes), with a fixed cost for each duplication and each loss; these reconciliations can be defined using just the first two conditions of Definition 1, with no eligibility matrix input, and where each $X_{i}\in \Delta$ has $|X_{i}|=1$. In this case, reconciliations can be easily found in linear time (Goodman *et al.*, 1979). Other models have considered horizontal transfers, merges/splits and shuffling events, but again with each duplication consisting of a single domain (Hallett *et al.*, 2004; Stolzer *et al.*, 2015; Yi-Chieh *et al.*, 2012). In contrast, Aluru and Singh (2020) and Dondi *et al.* (2019) consider reconcilations where single duplication events can consist of multiple units (e.g. domains or sequences), but they do not consider the positional information of these units and thus these duplications are not constrained to be tandem duplications; these formulations, which we term as Concurrent Duplication Loss (CDL) reconciliations, can be defined using all conditions except Condition 3c from Definition 1, and with no eligibility matrix as input. In Dondi *et al.* (2019), a maximum parsimony approach with fixed duplication and loss costs is used, it is shown that the CDL reconciliation problem is NP-complete, and a fixed-parameter tractable algorithm (along with its implementation MultRec) is given. In Aluru and Singh (2020), more general duplication cost functions based on duplication size are considered, and an ILP solution is given. While these previous models did not explicitly consider tandem duplications, a key insight of our work is that we can define TDL reconciliations with a simple adaptation of the definition of CDL reconciliations (Aluru and Singh, 2020; Dondi *et al.*, 2019) with the use of an eligibility matrix, which specifies which domain pairs can be part of the same tandem duplication. In the next section, we show how the elements of this key matrix are computed from input domain and gene trees and the relative positions of domains in modern day sequences. We note that previous approaches have also considered tandem duplications (Savard *et al.*, 2011), while additionally considering domain shuffling, but with the goal of counting the number of evolutionary events rather than assigning them to specific domains.

## 3 Identifying tandem duplication eligible domain pairs

In a tandem domain duplication, one or more consecutive domains present in a single gene are duplicated at once. In order to correctly identify tandem duplications, we need to be able to differentiate between a tandem duplication of multiple domains and multiple individual duplication events. This requires knowing the relative positions of a set of domains and their copies post-duplication. Specifically, suppose a gene contains *k* domains, 1,…,k. Let *P_i_* be the position of domain *i* in a left to right ordering of domains on the protein, and assume without loss of generality that $P_{i}<P_{i+1}\forall i$. Suppose domains *j* and *j *+* *1 are involved in duplication events, adding domains j′ and (j+1)′. We consider this as a tandem duplication if and only if after this has occurred, $P_{j}<P_{j+1}<P_{j\prime }<P_{(j+1)\prime }$ (see Fig. 1).

Unfortunately, information on the relative ordering of ancestral domains is lost in domain phylogenies. In order to infer these, we make the following assumptions:

1. When a tandem duplication occurs, the duplication copy is inserted immediately after the original domains. If a tandem duplication of size *k* occurs, affecting domains with positions i,i+1,…,i+k−1, then the copy of the domain with position *j* is inserted at position *j* + *k*. Any domain previously at position l>i+k−1 will have position *l* + *k* after the duplication.
2. Domain shuffling does not occur; that is, if at any time point we have *P_i_* < *P_j_* for domains *i* and *j*, then at no time point shall *P_j_* > *P_i_*. In the domain tree, this applies to all pairs of nodes *i* and *j*, and any of their descendants labeled with the same names (see Fig. 1).
3. Children of co-duplication nodes are identical to and maintain the same position as the parent.

The first and third assumptions are generally taken to be biologically reasonable, while the second may not be correct in all cases. Gene shuffling is known to occur in some families (Niimura and Nei, 2003), and may occur between domains as well. Our framework may not correctly identify tandem duplications in these cases. With these assumptions, we have a simple algorithm for determining whether a pair of ancestral domains could be involved in a tandem duplication. This algorithm additionally requires no losses after tandem duplications in order to guarantee correctness. Otherwise, necessary position information may be lost. We examine the case of domains duplicating within a single gene first, and then extend this solution to reconciliations with more than one gene.

### 3.1 Single gene case

In the single gene case, we examine a domain tree *D* that maps entirely to a single gene *g*. In the following, let *P_u_* be the actual position of domain node *u* in the first sequence it occurred in, while ${\hat{P}}_{u}$ is the position value we will infer. Note that because we have the positions of domains in modern day sequences, we can annotate each leaf *i* in the domain tree with an integer variable ${\hat{P}}_{i}$ that corresponds to the position of that domain within *g* (i.e. we set ${\hat{P}}_{i}=P_{i}$). In this case, these ${\hat{P}}_{i}$’s are distinct. Position values for internal nodes are set based on the values of their children. Let *u* be an internal domain node with children *v*, *w*. Following our assumption that the original copy of the domain is the leftmost one in the sequence, we set ${\hat{P}}_{u}=\min({\hat{P}}_{v},{\hat{P}}_{w})$. Note that this is an arbitrary decision, and we could instead have instead assumed that the original copy of the domain is the rightmost one. We use these position values to determine whether two nodes could be involved in a tandem duplication as follows:

Definition 2 (Pairwise Eligibility). *Let u and x be two internal nodes in domain tree D with children v, w and y, z*, *respectively. Suppose w.l.o.g. that* ${\hat{P}}_{v}<{\hat{P}}_{w},\;{\hat{P}}_{y}<{\hat{P}}_{z}$*, and* ${\hat{P}}_{v}<{\hat{P}}_{y}$*. We say that u and x are pairwise eligible if neither u nor x is an ancestor of the other, and* ${\hat{P}}_{v}<{\hat{P}}_{y}<{\hat{P}}_{w}<{\hat{P}}_{z}$.

Pairwise eligibility tells us whether two nodes could have been involved in a tandem duplication or not. Being pairwise eligible does not imply that two nodes were involved in a tandem duplication, but as we will see in the remainder of this section, in the absence of certain losses, two nodes that duplicate in tandem are guaranteed to be pairwise eligible. The notion of pairwise eligibility extends naturally from pairs of nodes to sets of nodes:

Definition 3 (Tandem Duplication Eligibility). *Let* $X=\{u_{1},\ldots ,u_{k}\}$  *be a set of two or more internal nodes in domain tree D. We say that X is tandem duplication eligible if u_i_ and u_j_ are pairwise eligible for all* i,j∈X,i≠j.

Like pairwise eligibility, sets of domains that underwent a tandem duplication event will be tandem duplication eligible in the absence of certain losses, but the converse is not necessarily true. We prove this in Theorem 1. Due to space considerations, all proofs are in the Supplementary Material. 

Theorem 1. *Let D be a domain tree containing all domains mapping to a single leaf gene. Let* $\left\{u_{1},\ldots ,u_{k}\right\}$  *be a set of internal nodes in D corresponding to domains involved in a tandem duplication, and suppose no losses occur among their descendants. Then, under assumptions (1) and (2)*, $\left\{u_{1},\ldots ,u_{k}\right\}$  *is tandem duplication eligible.*

### 3.2 Multi-gene case

In the multi-gene case, position values are no longer distinct. Each domain is assigned a position relative to other domains on the same gene, so domains appearing in different genes may have the same position value. The notion of pairwise eligibility is complicated by the fact that it is no longer clear whether two ancestral domains appear in the same gene without a mapping. Subtrees rooted at two internal domain nodes may not have any overlap in the genes that their leaves were present in. In these instances, we have no information on whether the domains at their root could be part of a tandem duplication. The key insight here is that we can only test tandem duplication eligibility between domains with descendants occurring in the same gene. Let *u* be an internal node in domain tree *D* with children *v* and *w*, and *g* be a leaf in gene tree *G*. We say that *g annotates u* if *D_v_* and *D_w_* both contain domains occurring in *g*. A gene *g* annotates a pair of domain nodes $\left\{u_{1},u_{2}\right\}$ if it annotates both *u*_1_ and *u*_2_. Any pair of internal nodes $\left\{u_{1},u_{2}\right\}$ in *D* may be annotated by zero, one or multiple genes. In each of these cases, we determine whether *u*_1_ and *u*_2_ are pairwise eligible as follows:

- No annotations. If *u*_1_ and *u*_2_ have no shared annotations, then we cannot determine the relative ordering of their children. That is, we cannot determine whether *u*_1_ and *u*_2_ are pairwise eligible, and so we are permissive and say that *u*_1_ and *u*_2_ are pairwise eligible.
- One annotation. If *u*_1_ and *u*_2_ have one shared annotation, then there exists some leaf gene *g* that annotates both *u*_1_ and *u*_2_. Let D[g] be the induced subtree of *D* formed from the leaves of *D* present in gene *g*; that is, D[g] corresponds to the subtree of *D* rooted at the least common ancestor of the domains of gene *g*. Note that *u*_1_ and *u*_2_ are both preserved in D[g]. We say *u*_1_ and *u*_2_ are pairwise eligible if they are pairwise eligible in D[g] according to the single gene tree case.
- Multiple annotations. Suppose *u*_1_ and *u*_2_ are annotated by genes $g_{1},\ldots ,g_{k}$. Nodes *u*_1_ and *u*_2_ are pairwise eligible if they are pairwise eligible in $D[g_{i}]$ for any *g_i_*.

See Supplementary Figure S4 for an illustration of each case. As in the single gene case, we say that a set $\left\{u_{1},\ldots ,u_{k}\right\}$ of two or more internal domain nodes is *tandem duplication eligible* if *u_i_* and *u_j_* are pairwise eligible for all i,j∈[1,k].

Theorem 2. *Let D be a domain tree, and G be the containing gene tree. Let* $\left\{u_{1},\ldots ,u_{k}\right\}$  *be a set of internal nodes in D mapped to gene node g in G corresponding to domains involved in a tandem duplication. Suppose there exists some* $g_{i}\in L(G_{g})$  *such that no losses occur in the domain subtrees of* $D[g_{i}]$  *rooted at* $u_{1},\ldots ,u_{k}$*. Then, under assumptions (1), (2) and (3)*, $\left\{u_{1},\ldots ,u_{k}\right\}$  *is tandem duplication eligible.*

Theorem 2 tells us that if a tandem domain duplication occurred, then the nodes in the domain tree representing those domains will be marked as pairwise eligible. Of course, the converse is not true; there may be some sets of nodes which are tandem duplication eligible but were not tandem duplications.

Theorem 3. *Suppose nodes u and v in domain tree D are mapped to gene g and annotated by genes* $g_{1},\ldots ,g_{k}$*, and no losses occur in their subtrees D_u_ and D_v_. If u, v are marked as duplication nodes by the reconciliation framework and are pairwise eligible in* $D[g_{i}]$  *for any g_i_, then u_1_, u_2_ are pairwise eligible in* $D[g_{i}]$  *for all g_i_.*

Theorem 3 tells us that with no losses and under our assumptions, genes will not ‘disagree’; that is, if domains are ordered on one gene such that their ancestors could have been tandem duplications, assumption (2) tells us that sibling domains must be ordered the same way on any other gene as well. In cases where genes do disagree, either assumption (2) has been violated, or our domain tree is incorrect. In such cases, we do not constrain tandem duplication eligibility.

For a domain tree with *n* nodes, we define the *n *×* n eligibility matrix E* such that *E_ij_* = 1 if domains *i* and *j* are pairwise eligible, and 0 otherwise. Note that $E_{ii}=0$  ∀i by the pairwise eligibility definition. The eligibility matrix is used to weed out pairs of domains that cannot be part of the same tandem duplication. While it is conservative in allowing some false positive pairs, in practice we see that our approach is dramatically more accurate than approaches that do not constrain tandem duplications.

In this section, our proofs relied on having no losses following duplications. This may seem like a serious limitation, but our method can maintain accuracy even when some losses occur. First, recall that the position label of any internal node *u* of our gene tree is taken from the leaf in its subtree with the minimum position value. If any other leaf in the subtree is lost, *u* will still be assigned the correct position value. Second, if a tandem domain duplication occurs in an ancestral gene *g*, then the descendants of those domains will be passed to all descendants of that gene. If no losses occur among those descendant domains in even one gene in *L*(*g*), our method will mark the domains involved in that duplication event as tandem duplication eligible (see Supplementary Fig. S3). Taken together, these points make our method robust to losses even in relatively high loss regimes, as we will show via simulations in Section 5.2.

## 4 A heuristic solution

Once the eligibility matrix is computed, the TDL reconciliation problem can be solved with a simple modification to the ILP we previously introduced (Aluru and Singh, 2020) to solve the CDL reconciliation problem. Solving the TDL reconciliation problem with this ILP requires the addition of a single constraint (see Supplementary Material for the full ILP solution). However, this solution requires $O(m^{2}n^{2})$ constraints on an instance with *m* genes and *n* domains, making it computationally infeasible for large gene families with many domains. To scale to larger instances, we introduce a heuristic which works by breaking up the original problem into a series of sub-problems, and then running a simpler ILP on each sub-problem. In Section 5, we show that this heuristic infers tandem duplications almost as well as the original ILP, while being significantly faster. Our full ILP solves the TDL reconciliation problem exactly; however, note that we do not add constraints to prevent non-consecutive domains in tandem duplications, or non-consecutive tandem duplications. In practice, these events may arise if the input contains errors or violates the assumptions given in Section 3.

To decompose our ILP, note that all domains in any tandem duplication set must be mapped to the same gene node. Similarly, losses can only occur on edges in the domain tree between domain nodes mapped to different gene nodes. This means that once a mapping is fixed, the number of losses can be immediately calculated, and duplication sets can be constructed by looking individually at the sets of domains mapped to each gene tree node. In other words, given a mapping oracle which gives the optimal full mapping $\gamma^{*}$, we can find the optimal tandem duplication sets for this mapping by running a reduced version of our ILP on the domain sets mapped to each gene node. In the remainder of this section, we describe our heuristic, which consists of three steps:

1. Map domain nodes to gene nodes using an oracle that gives an ‘imperfect’ but valid mapping.
2. Isolate sets of domains mapped to each gene node and infer tandem duplications within each gene using a reduced ILP.
3. Remap domains to genes by moving tandem duplication sets until convergence.

Each of these steps is described in more detail next, and an overview of our entire approach is given in Supplementary Figure S2.

### 4.1 Mapping oracle

For our mapping oracle, we use the mapping from a simpler problem, the DL reconciliation problem. In this variant, tandem duplications are not considered, and the only events allowed are single domain duplications and losses (see Section 2.2). This problem can be solved in O(|D|) time using an LCA full mapping *γ* (Goodman *et al.*, 1979):

-
γ(u)=σ(u) ∀u∈L(D).
-
$\gamma (u)=\mathrm{LCA}(\gamma (u_{1}),\gamma (u_{2}))$ ∀u∈I(D) with children *u*_1_, *u*_2_.

This mapping may be suboptimal for the full TDL reconciliation problem. However, as we show in Section 5.2, we find in simulation that this mapping tends to be very close to the ground truth mapping.

### 4.2 Reduced ILP

Given a full mapping of domains to genes, we need to assign events to each internal domain. As mentioned earlier, only domains mapped to the same gene may be involved in a tandem duplication. This means that when determining tandem duplication events within a gene, we can ignore all domains not present in that gene. Let *R*(*g*) be the set of all domains mapped to gene *g*. We find the sequence of tandem duplication events that minimizes the reconciliation parsimony cost for these nodes. This is done using a reduced ILP, given below, which is based on our full ILP (given in the Supplementary Material). In our reduced ILP, we remove mapping constraints, keeping only duplication/co-duplication constraints. We first define constants that encode the inputs:

1. *d_uv_* for each pair of nodes u,v∈R(g). We fix *d_uv_* = 1 if $u\geq_{D}v$ and 0 otherwise. That is, *d_uv_* encodes ancestry relationships between nodes in the domain tree.
2. *e_uv_* for each pair of nodes u,v∈R(g). We set *e_uv_* = 1 if *u*, *v* are eligible to be in the same tandem duplication set according to the eligibility matrix *E*.
3. *b_u_* for each node u∈R(g). We set *b_u_* = 1 if *u* must be part of a tandem domain duplication according to rule 2 of the TDL problem using the oracle’s node mapping.

Next, we define our variables, which assign events to domain nodes:

1. *X_uv_* for each pair of nodes u,v∈R(g), u≠v. *X_uv_* will be set to 1 if nodes *u* and *v* are found to be part of the same tandem duplication; that is, *X_uv_* = 1 if there is a set a∈Δ such that u,v∈a.
2. *T_uk_* for each node u∈R(g) and $1\leq k\leq K_{\max}$, where *K*_max_ is the maximum allowed size of a tandem duplication. *K*_max_ can be set to half the number of domain nodes in *R*(*g*) to allow for all possible tandem duplication sizes. *T_uk_* will be set to 1 if the length of the tandem duplication that node *u* belongs to is *k*, and 0 otherwise.

Our reduced ILP is given below. Because there are no losses between domains mapped to the same gene, the objective function only needs to minimize the cost from tandem duplication events. This cost is given by $c_{D}\left[\sum_{u}b_{u}-\sum_{u,k}\left(\frac{k-1}{k}T_{uk}\right)\right]$, with the *b_u_* term counting the number of nodes taking part in tandem duplication sets and the *T_uk_* term ensuring we do not double count tandem duplication events. The objective function omits the $\sum_{u}b_{u}$ term since this is a constant, and instead maximizes the negative of the second term. The constraints given are a direct translation of rules 3 and 4 from the TDL reconciliation problem. The first constraint ensures that two comparable domains may not be in the same tandem duplication set. The second constraint enforces rule 4 of problem definition, ensuring temporal consistency between tandem duplications. The third and fourth constraints ensure our sets are consistent, requiring that if *u* is marked in a tandem duplication set with *v*, then *v* must also be in a set with *u*. Similarly, if *u* and *v* are part of a tandem duplication set, and *v* and *w* are also in a tandem duplication set, then *u* and *w* must also be part of the same set. Constraints 5 and 6 make sure that nodes are only part of tandem duplication sets if they are duplications to begin with, and that two domains may be part of the same tandem duplication set only if they are tandem duplication eligible. Constraints 7 and 8 ensure the *T* variables accurately count the size of tandem duplications, for the purpose of cost calculation. Finally, constraint 9 is the integer constraint on the *X* and *T* variables.

$$\begin{array}{c} \max\;c_{D}\sum_{u,k}\left(\frac{k-1}{k}T_{uk}\right)\;\;s.t.\\ X_{uv}\leq 1-d_{uv}\;\;\forall u,v\in R(g))\\ X_{uv}\leq (1-X_{yz})+(1-d_{uy}d_{zv})\;\;\forall u,v,y,z\in R(g)\\ X_{uv}\geq X_{uy}+X_{vy}-1\;\;\forall y,u,v\in R(g),u\neq v\\ X_{uv}=X_{vu}\;\;\forall u,v\in R(g)\\ X_{uv}\leq b_{u}\;\;\forall u,v\in R(g)\\ X_{uv}\leq e_{uv}\;\;\forall u,v\in R(g)\\ \sum_{0\leq k\leq K_{\text{max}}}T_{uk}\leq 1\;\;\forall u\in R(g)\\ T_{uk}\leq \frac{1}{k}(b_{u}+\sum_{v\neq u}X_{uv})\;\;\forall u\in R(g),1\leq k\leq K_{\text{max}}\\ X_{uv},T_{uk}\in \{0,1\}\;\;\forall u,v\in R(g),1\leq k\leq K_{\text{max}}. \end{array}$$

If *R*(*g*) is accurate, the tandem duplication events yielded by this process will be part of an optimal solution to the TDL reconciliation problem, and repeating this process on all nodes g∈G gives all tandem duplication sets in the optimal TDL solution.

### 4.3 Node remapping

Once all domains are mapped and assigned events, we attempt to improve our solution by remapping domain nodes when doing so reduces the reconciliation cost. Note that our initial mapping was an LCA mapping. This means that for any domain node *u* initially mapped to γ(u), the only valid genes to which *u* could be mapped are ancestors of γ(u). The following lemma shows the effect of such a remapping move on the reconciliation cost. It has been proven several times (e.g. see Aluru and Singh, 2020; Dondi *et al.*, 2019), and we reproduce it here without proof:

Lemma. *Let G, D and γ be a gene tree, domain tree and full mapping*, *respectively. Let* u,v,w∈D  *such that* $u=p_{D}(v)=p_{D}(w)$*. Let* i=γ(u)  *and* j=LCA(γ(v),γ(w))  *and suppose* $i{>}_{G}j$*. Then, at least* $d_{G}(i,j)$  *losses can be saved by setting* γ(u)=j.

This lemma tells us that the LCA mapping minimizes the number of losses inferred. Remapping a domain to an ancestor will increase the number of losses and therefore the overall cost of the reconciliation. The only case in which this is acceptable is if remapping a node can also decrease the number of tandem duplications we infer.

Remapping is done as follows. First, we create an ordering over tandem duplications sets such that for any two sets *X* and *Y*, if ∃x∈X,y∈Y s.t. $x{<}_{D}y$, then *X* occurs after *Y* in the ordering. We note that if there exists such *x* and *y*, then there cannot exist nodes u∈X,v∈Y s.t. $v{<}_{D}u$, or else constraint (4) of the TDL reconciliation problem would be violated. Roughly, this creates a ‘top down’ traversal over tandem duplication sets. We process tandem duplications in this order. For any inferred tandem duplication set *X* occurring in gene *g*, we check whether every domain node in *X* can be remapped to an ancestor of *g* without violating any conditions in the definition of a TDL reconciliation. For any ancestor $g^{*}$ to which *X* can be remapped, we check whether *X* can be combined with an existing tandem duplication set already mapped to $g^{*}$. If so, the change in cost from remapping all nodes in *X* to $g^{*}$ is $c_{L}\times |X|\times d_{G}(g,g^{*})-c_{D}$. If there exists some $g^{*}$ for which the change in cost is negative, we remap all domains in *X* to the node $g^{*}$. Prior to remapping, our solution was guaranteed to have the minimum possible number of tandem duplication sets subject to the oracle’s mapping. If a duplication set *X* originally mapped to gene *g* is remapped to an ancestor node $g^{*}$, we know that the tandem duplication sets mapped to gene *g* are still optimal. Similarly, because the number of tandem duplications in $g^{*}$ is not allowed to increase from remapping, the tandem duplications at $g^{*}$ after this operation are also optimal. Therefore, we do not need to rerun our ILP after this step. We repeat the remapping step until convergence. The final node mappings and tandem duplication sets are the output of our heuristic.

## 5 Results

### 5.1 Simulation

We tested our methods on both simulated and real datasets. Simulated data were generated using our TreeSim package, first described here (Aluru and Singh, 2020), and with full details provided in the Supplementary Material. Briefly, we generate simulated gene tree topologies, and generate domain tree topologies within them. Gene trees are generated as randomized topologies with a fixed number of leaves. The overall branch length on a root to leaf path in the gene tree represents the expected number of sequence mutations that occur along that branch. This is taken as an input to our simulation. Domain trees are simulated with respect to a given gene tree. Co-duplication events occur at each gene tree bifurcation. Between these events, tandem domain duplications and losses occur according to a birth–death process. The distance between events follows an exponential distribution whose expected value is given as an input parameter. Loss events affect any single domain, whereas tandem duplications involve one or more consecutive domains at once. Duplications are inserted immediately after the original copies. We set the probability of a tandem duplication of size *k* occurring to be $1/2^{k}$. This makes single duplications the most likely type, while making tandem duplications of size greater than three rare. No other events are simulated. The ratio of duplication to loss events is variable, tuned to create a series of tandem duplications followed by a high probability of repeated losses. We note that this represents a worst case scenario for our methods as losses may violate the assumptions necessary to accurately infer whether domains are eligible to be in the same tandem duplication. As we show next, however, our methods have excellent performance despite this, and will likely only be better on real world datasets.

### 5.2 Simulation results

Simulations were run using gene trees with eight leaf nodes and an average root to leaf distance of 0.3. We varied the mean distance between evolutionary events in *D* between 0.1 and 0.01 to examine its effects on reconciliation accuracy. Large evolutionary event distances produce one or two events per gene, while low event distances can simulate a cascade of consecutive domain events within a single gene. We generate 50 gene and domain tree topologies at each of the five event distances, giving a total of 250 simulations. On average, we obtain 126, 83, 55, 29 and 17 leaf domains for event distances of 0.01, 0.025, 0.5, 0.75 and 0.1, respectively. For these event distances, respectively, we have on average 20, 7, 3, 1 and 0 domain losses, and 34, 18, 11, 5 and 2 tandem duplication events, of which roughly half affect two or more domains. All tests were performed using the trees generated by our simulation.

We first test the accuracy of several methods that map domains to genes, as we need to use one of these as the mapping oracle for our heuristic. We test LCA mapping as well as the mapping output by a previous algorithm, MultRec (Dondi *et al.*, 2019), which solves the CDL reconciliation problem. Although using it as a mapping oracle would defeat the purpose of our heuristic, we test the accuracy of the mapping output by our full ILP for comparison purposes. For each of these three methods, we give it a simulated gene and domain tree, and mapping accuracy is measured as the fraction of domain nodes that are mapped to the correct gene node. For each tested method, Table 1 shows the average mapping accuracy for each event distance. We find that for all methods accuracy is nearly perfect for all tested event distances. The LCA mapping, which is both the simplest and fastest method, is slightly better at the lowest event distances. Therefore, we use this method as the mapping oracle for our heuristic.

**Table 1.** Accuracy of mappings obtained by the LCA method, our full ILP and MultRec

	Event distance				
	0.010	0.025	0.050	0.075	0.100
LCA	0.993	1.000	1.000	1.000	1.000
Full ILP	-	0.999	1.000	1.000	1.000
MultRec	0.990	0.997	1.000	1.000	1.000

*Note*: For each event distance, we give the average mapping accuracies obtained across simulations when using mappings obtained by each of three methods. All three methods have nearly perfect performance in all instances, indicating that in most cases, using mappings from the fastest method (LCA) is sufficient. We omit results for the ILP at an event distance of 0.01 because it was unable to run in a reasonable time.

**Fig. 2.**
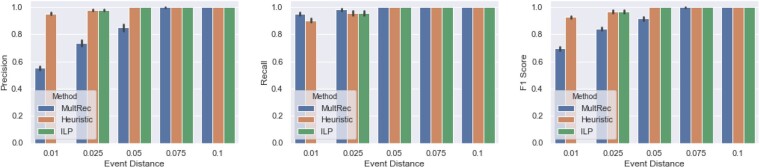
Performance of methods in inferring tandem domain duplications. Average precision (left), recall (middle) and F1-scores (right) of MultRec (blue), our heuristic (orange) and our full ILP (green) when run on 50 simulations each at the five given event distances. Error bars depict one standard deviation around the mean. At high event distances, all three methods have excellent precision, recall and F1-scores. At lower event distances, the heuristic and ILP have significantly higher precision than MultRec but slightly lower recalls. The heuristic and ILP also have significantly higher F1-scores than MultRec at low event distances. While due to runtime, we do not report results for the ILP at an event distance of 0.01, we observe that the heuristic achieves perfect scores at high event distances and is only slightly worse than the ILP at an event distance of 0.025.

Next, we assess the ability of our full ILP, our heuristic and MultRec to infer tandem duplications. Each method is given the domain and sequence trees generated by a simulation, and our methods are additionally given an eligibility matrix, computed using these trees and the positions of domains in the generated extant sequences as described in Section 3. For each run of each of these algorithms, we compute precision, recall and F1 scores as follows. For every tandem duplication set *X* found by a method, we create a set consisting of all pairs of domains (*a*, *b*) such that a,b∈X. The tandem duplication sets are combined into one set *Pred* of pairs of domains predicted to be part of the same tandem duplication. We create a similar set *Real* of pairs of domains that are actually part of the same tandem duplication in the simulation. Precision is computed as |Pred∩Real|/|Pred| and recall is computed as |Pred∩Real|/|Real|. F1-scores, which compute the tradeoff between precision and recall, are computed as $2\cdot \frac{\text{Precision}\cdot \text{Recall}}{\text{Precision}+\text{Recall}}$. Figure 2 compares the precision, recall and F1-scores of our methods to MultRec. At low event distances, our methods have significantly better precisions than MultRec; our average precisions are 1.73, 1.33 and 1.18 times higher than MultRec’s at event distances of 0.01, 0.025 and 0.5, respectively. On the other hand, MultRec has slightly better recall than our methods, though for all distances, its average recall is <5% better. Across event distances, our approaches have excellent performance with average precisions ≥0.95 and average recalls ≥0.9. Furthermore, our methods have significantly higher F1-scores than MultRec at event distances up to 0.05. By incorporating tandem duplications into our model, our method is able to significantly reduce the number of false positive tandem duplication sets found, while removing only a small number of true positives relative to MultRec. True positives can be removed by our approach when domain losses lead to the mislabeling of domain pairs as tandem duplication eligible. Moreover, our heuristic approach detects tandem duplications almost as well as our exact ILP.

We also report runtimes of our approaches. Each of the methods was run on a 3.2 GHz eight core Xeon processor with 64 GB of RAM. We used the gurobi (LLC Gurobi Optimization, 2021) package to solve all ILPs. Table 2 shows the average runtime across the simulations of our heuristic and ILP approaches, as well as of MultRec. Our full ILP does not scale well as event distance decreases, and at the smallest event distance of 0.01 did not finish running on our 50 simulations in a reasonable time. In contrast, the heuristic approach scales well across event distances. At the lowest event distance of 0.01, it takes about 4 s per example on average, while providing significantly better tandem duplication sets than MultRec.

**Table 2.** Average runtime in seconds of our heuristic, our full ILP and the MultRec program

	Event distance				
	0.010	0.025	0.050	0.075	0.100
Heuristic	4.041	1.369	0.48	0.13	0.062
Full ILP	-	282.734	35.214	1.706	0.333
MultRec	0.975	0.185	0.041	0.018	0.012

*Note*: For each event distance, the average runtime in seconds across 50 simulations is reported. Our heuristic scales nearly as well as MultRec while providing significantly better tandem duplication inference. In contrast, at event distance 0.01, the ILP did not finish running on the 50 simulations in under 24 h.

### 5.3 Results on a biological dataset

Having shown that our heuristic works well in identifying tandem repeats on simulated data, we next apply it to a protein sequence family with numerous repeated domains. In particular, we used our heuristic to analyze the evolutionary history of filamin domains from the Filamin-A protein family. Light *et al.* (2012) analyzed this family in detail and found via manual inspection of domain sequence similarity that although long tandem repeats of filamin domains are a hallmark of this protein family, most sequences do not exhibit evidence of tandem duplications. In particular, two species, *Hirudo medicinalis* and *Trichoplax adhaerens*, contain lineage-specific expansions in their filamin domain arrays. However, while the Filamin-A protein of *T.adhaerens* shows clear signs of tandem duplications, in *H.medicinalis* this protein appears to have increased its domain repertoire through repeated single domain duplications. We sought to recapitulate these results in a fully automated manner.

**Fig. 3.**
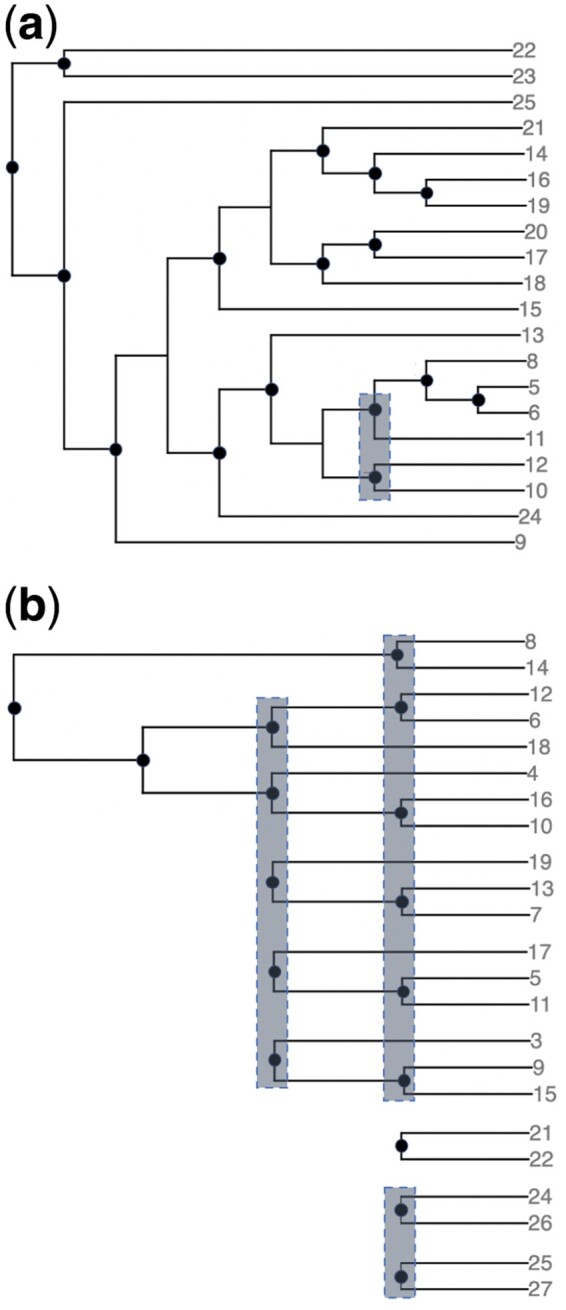
Lineage-specific filamin domain expansions in Filamin-A proteins. Shown are clades of domains whose leaves consist of domains found only in (**a**) *Hirudo medicinalis* or (**b**) *Trichoplax adhaerens*. These subtrees of the full domain tree represent lineage-specific expansions in each species. The number labeling each leaf refers to the relative position of each domain in the constituent protein. Gray rectangles specify the tandem duplication events inferred by our heuristic approach. All internal nodes not covered by these gray rectangles correspond to single domain duplications. Branch lengths are not representative. In *H.medicinalis*, we see a series of single duplications, with one small tandem duplication in the middle. On the other hand, *T.adhaerens* exhibits a very clear pattern of tandem duplications. Models that attempt to minimize the number of tandem duplications without considering domain positions would be mostly correct for (b), but not for (a). With our model, we can accurately infer duplication events in both scenarios.

We built a dataset of 18 Filamin proteins from 18 organisms (Supplementary Table S1), extracted all filamin domains from these sequences using HMMER (Eddy, 2011) and built a multiple sequence alignment of these domains using Clustal Omega (Sievers and Higgins, 2018). These proteins contain from 5 to 36 filamin domains per sequence. We inferred a domain tree using RAxML (Stamatakis, 2014), and used the gene tree given in Light *et al.* (2012). We ran our heuristic to find tandem duplications, and confirmed the existence of a cascade of tandem duplications in *T.adhaerens* while finding mainly single duplications in the lineage-specific expansion of *H.medicinalis* (see Fig. 3). Unfortunately, MultRec was unable to run on these sequences within 6 h but the CDL model would mark both expansions as a series of tandem duplications whereas DL reconciliation would mark both as repeated single duplications. In contrast, by explicitly modeling tandem duplications, our approach is able to distinguish between these very different evolutionary patterns that can occur in real sequence families.

## 6 Conclusion

In this work, we introduce an approach to infer the evolutionary history of repeat domains where we explicitly model tandem domain duplications, a frequent evolutionary event. We demonstrate how to use domain and gene trees, along with the positions of domains within extant genes, to identify whether domains can be within the same tandem duplication. We have proved the correctness of this, under the assumptions that tandem duplication copies occur immediately after (or equivalently before) the original domains, domain shuffling does not occur, and that losses do not occur to domains that were involved in a tandem duplication. We use this knowledge to constrain allowable tandem duplications found by reconciling domain and gene trees, and give both an exact ILP solution and a fast yet effective heuristic to infer these reconciliations. We note that our approach may allow tandem duplications consisting of sets of domains that are not adjacent to each other; this can occur when losses follow tandem duplications or there are errors in the input trees. Theoretically, it remains possible that using our method for determining whether domains can be part of the same tandem duplication, optimal solutions for TDL reconciliation may include tandem duplication sets consisting of non-contiguous domains even when evolutionary events occurred only under the assumptions of our model; however, we have not observed such an example. Despite these caveats, we show via extensive testing that the algorithms we develop significantly improve our ability to detect and distinguish between real tandem duplications and repeated single duplications. Moreover, we show the importance of this distinction using a protein family that exhibits both types of duplications in separate lineages.

While we focused on three types of evolutionary events—co-duplications, tandem duplications and losses—other types of events, including horizontal domain transfers and domain merge/split events have been previously modeled. Adding these events to the TDL reconciliation framework will increase the accuracy of event inference in gene families where they occur. Modeling domain shuffling events, in which domains swap positions on the protein, would be particularly useful to our model as we rely on the inference of ancestral domain positions.

With large protein families, or those with many domains, runtime can be an issue. This is especially important in families with tandem duplications, whose existence implies a large number of domains per protein. Our ILP solution, while exact, is unable to scale to realistically sized protein and domain trees. Our heuristic mitigates this issue, speeding up the process significantly while maintaining accuracy by running a simplified ILP on a series of smaller sub-problems. This approach scales to tens of proteins with dozens of domains, but has difficulty with protein families of interest such as the nebulin and immunoglobulin families, which can have over 100 domains per protein. Developing faster heuristics to assign tandem duplication sets would allow these families to be analyzed. Another challenge in inferring the evolutionary history of repeat domains within a protein family is that these domains can be quite short, with some containing as few as 20 amino acids. In these cases, it is difficult to obtain accurate phylogenies using traditional methods like RAxML. Recent methods such as TreeFix (Wu *et al.*, 2013) mitigate this issue by balancing both maximum likelihood and duplication-loss reconciliation scores. Using TDL-derived reconcilation scores may improve the accuracy of these approaches, as they will correspond to higher quality reconciliations.

An especially exciting avenue for future work is the large-scale application of our approach to other orthogroups, either in the context of domain and gene tree reconciliations or gene and species tree reconciliations. We hypothesize that large tandem duplications of certain domains and genes will map to places in the phylogeny where accelerated evolution leads to important functional innovations, and anticipate that the methods we have developed in this article will be a great aid in pinpointing these important events.

*Data Availability:* The code underlying this article is available at https://github.com/Singh-Lab/TandemDuplications

*Funding Sources:* This work was supported by the National Institute of Health (NIH) [grant number R01-GM076275 to MS] and National Science Foundation (NSF) [grant number ABI-1458457 to MS]

*Conflict of Interest*: none declared.

## Supplementary Material

btab329_Supplementary_Data
