# Supplementary material for: Improved inference of tandem domain duplications
Source: Bioinformatics. 2021 Jul 12;37(Suppl 1):i133–41. doi: 10.1093/bioinformatics/btab329 (PMC8275333; doi:10.1093/bioinformatics/btab329)
Supplement: btab329_Supplementary_Data [file btab329_supplementary_data.pdf]

Supplement

Chaitanya Aluru, Mona Singh

January 2021

A ILP Solution

Here we present the full ILP solution to the TDL Reconciliation Problem. Our solution is very similar to the ILP we presented in [1] to solve the Concurrent Duplication Loss (CDL) Problem, but with the addition of tandem duplication eligibility constraints. We take as input a gene tree G, a domain tree D, a leaf mapping $\sigma : L(D) \to L(G)$, and a $|V(D)| \times |V(D)|$ tandem duplication eligibility matrix E. We encode the inputs into the following constants:

1. g_{uv} for each pair of nodes $u, v \in V(G)$. We fix $g_{uv} = 1$ if $u \geq_G v$ and 0 otherwise. That is, g_{uv} encodes ancestry relationships between nodes in the gene tree.
2. d_{uv} for each pair of nodes $u, v \in V(D)$. We fix $d_{uv} = 1$ if $u \geq_D v$ and 0 otherwise. That is, d_{uv} encodes ancestry relationships between nodes in the gene tree.
3. e_{uv} for each pair of nodes $u, v \in V(D)$. We set $e_{uv} = 1$ if u, v are eligible to be in the same tandem duplication set according to the eligibility matrix E.
4. $dist_T(u, v)$ for comparable nodes $u, v \in V(T)$, which corresponds to the distance in the tree T from node u to node v. We use distances for both G and D.

We encode the mapping and event assignments as a series of binary variables which the ILP optimizes over. Note that mapping variables are fixed for all $u \in L(D)$.

1. M_{ui} for each pair of nodes $u \in V(D)$ and $i \in V(G)$. The variables M_{ui} define the mapping γ in the reconciliation and will be set to 1 if $\gamma(u) = i$ and 0 otherwise. For $u \in L(D)$, for all $i \in V(G)$, M_{ui} is fixed according to the input leaf mapping; that is, it is set as 1 if $\sigma(u) = i$ and 0 otherwise.
2. Y_{uvij} for nodes $u, v \in V(D)$, $i, j \in V(G)$. Y_{uvij} is an indicator variable, and will be set to 1 if $\gamma(u) = i$ and $\gamma(v) = j$.
3. B_u for each node $u \in I(D)$. The variable B_u will be set to 1 if u is assigned to a tandem duplication set.
4. X_{uv} for pair of nodes $u, v \in I(D)$, $u \neq v$. X_{uv} will be set to 1 if nodes u and v are part of the same tandem duplication; that is $X_{uv} = 1$ if there is a tandem duplication set $a \in \Delta$ such that $u, v \in a$.

5. T_{uk} for all nodes $u \in I(D)$ and $1 \le k \le K_{max}$, where K_{max} is the maximum allowed size of a tandem duplication. K_{max} can be set to $|L(D)/2|$ and this allows all possible tandem duplication sizes. T_{uk} will be set to 1 if the size of the tandem duplication set that node u belongs to is k, and 0 otherwise. If u is not a member of a tandem duplication set, then $\forall k, T_{uk} = 0$

Our ILP is shown in Figure S1. Our objective function and constraints are a direct translation of the cost function and constraints given in Section 2.2 of the main paper.

$$\min \quad c_D \left[\sum_u B_u - \sum_{u,k} \left(\frac{k-1}{k} T_{uk} \right) \right] + c_L \sum_{\substack{u \in I(D) \\ v\ s.t.\ p(v)=u \\ i,j \in G}} dist_G(i,j) Y_{uvij} - 2c_L \sum_{u \in I(D)} (1 - B_u)$$

$$\begin{aligned}
\text{s.t.} \quad & \sum_j M_{uj} = 1 & \forall u \in V(D) && (1) \\
& M_{ui} \le (1 - M_{vj}) + g_{ij} & \forall u \in V(D), i,j \in V(G), v\colon p_D(v) = u && (2) \\
& Y_{uvij} \ge M_{ui} + M_{vj} - 1 & \forall u,v \in V(D), i,j \in V(G) && (3) \\
& Y_{uvij} \le M_{ui} & \forall u,v \in V(D), i,j \in V(G) && (4) \\
& Y_{uvij} \le M_{vj} & \forall u,v \in V(D), i,j \in V(G) && (5) \\
& B_u \ge \frac{1}{2} Y_{vwij}(g_{ij} + g_{ji}) & \forall u \in I(D), i,j \in V(G), u \text{ parent of } v,w && (6) \\
& X_{uv} \le 1 - d_{uv} & \forall u,v \in I(D) && (7) \\
& X_{uv} \le e_{uv} & \forall u,v \in V(D) && (8) \\
& X_{uv} \le \sum_i Y_{uvii} & \forall u,v, \in I(D), i \in V(G) && (9) \\
& X_{uv} \le (1 - X_{yz}) + (1 - g_{uy} g_{zv}) & \forall u,v,y,z \in I(D) && (10) \\
& X_{uv} = X_{vu} & \forall u,v \in I(D) && (11) \\
& X_{uv} \le B_u & \forall u,v \in I(D) && (12) \\
& X_{uv} \ge X_{uy} + X_{vy} - 1 & \forall y,u,v \in I(D), u \ne v && (13) \\
& \sum_{0 \le k \le K_{max}} T_{uk} \le 1 & \forall u \in I(D) && (14) \\
& T_{uk} \le \frac{1}{k}(B_u + \sum_{v \ne u} X_{uv}) & \forall u \in I(D), 1 \le k \le K_{max} && (15) \\
& M_{ui}, Y_{uvij} \in \{0,1\} & \forall u,v \in V(D), i,j \in V(G) && (16) \\
& B_u, X_{uv}, T_{uk} \in \{0,1\} & \forall u,v \in I(D), 1 \le k \le K_{max} && (17)
\end{aligned}$$

Figure S1: ILP Solution to the TDL Reconciliaton Problem

B Proofs

In this section, we provide proofs of the lemmas and theorems in Section 3 of the main paper.

Lemma 1. *If $P_u < P_v$, and no losses occur to descendants of u, then under assumptions (1) and (2), $\hat{P}_u < \hat{P}_v$.*

Proof. This is equivalent to showing that $\min_{u_i \in L(D_u)} \hat{P}_{u_i} < \min_{v_i \in L(D_v)} \hat{P}_{v_i}$. To do this, we examine the positions of u and v's descendants after duplication and loss events. Let u_1 and u_2 be the children of node u, and suppose that $P_{u_1} < P_{u_2}$. In other words, u_1 is the original domain while u_2 is the duplicated copy. From assumption 1, we see that immediately after duplication, $P_{u_1} = P_u$, and this implies $P_{u_1} < P_v$ while $P_{u_2} > P_u$. We see that at least one child of u will occur before v, and by similar logic no child of v will have a position value less than P_v. By assumption 2, domains may not swap position, so this will hold throughout the evolutionary history of the sequence of domains. So, if only duplications occur, then $\min_{u_i \in L(D_u)} \hat{P}_{u_i} < \min_{v_i \in L(D_v)} \hat{P}_{v_i}$. Even if losses occur, as long as no descendant of u is lost, the previous statement will hold as a loss in the subtree of v can only increase the value of $\hat{P}_v$ and losses of other domains cannot swap the relative order of any two domains. □

Theorem 1. *Let D be a domain tree containing all domains mapping to a single leaf gene. Let $\{u_1, \ldots, u_k\}$ be a set of internal nodes in D corresponding to domains involved in a tandem duplication, and suppose no losses occur among their descendants. Then, under assumptions (1) and (2), $\{u_1, \ldots, u_k\}$ is tandem duplication eligible.*

Proof. To show this, we must show that any two nodes u_i and u_j in the set are pairwise eligible. Suppose w.l.o.g. that $P_{u_i} < P_{u_j}$, and let nodes a, b be children of u_i with $P_a < P_b$ and c, d be children of u_j with $P_c < P_d$. By assumption 1, we know that immediately after the tandem duplication event, $P_a < P_c < P_b < P_d$. By lemma 1, this means that $\hat{P}_a < \hat{P}_c < \hat{P}_b < \hat{P}_d$. Therefore, u_i and u_j are pairwise eligible for all pairs, and $\{u_1, \ldots, u_k\}$ are tandem duplication eligible. □

Theorem 2. *Let D be a domain tree, and G be the containing gene tree. Let $\{u_1, \ldots, u_k\}$ be a set of internal nodes in D mapped to gene node g in G corresponding to domains involved in a tandem duplication. Suppose there exists some $g_i \in L(G_g)$ such that no losses occur in the domain subtrees of $D[g_i]$ rooted at $u_1, \ldots, u_k$. Then, under assumptions (1), (2) and (3), $\{u_1, \ldots, u_k\}$ is tandem duplication eligible.*

Proof. First, note that if g is a leaf node, then Theorem 1 applies and $\{u_1, \ldots, u_k\}$ is tandem duplication eligible. If g is an internal node, then we examine the positions of domains in genes on the path from g to g_i. Let u_i, u_j be any two domain nodes in the set of tandem duplication nodes such that $P_i < P_j$. We examine their subtrees in $D[g_i]$. If no losses occur, then only duplication and co-duplication events are present in $D[g_i]$. Because co-duplication events pass one copy of each domain present in a gene to each of its children, they appear as nodes with only a single child in $D[g_i]$. By assumption 3, the order of domains is preserved in co-duplications, so these events can be effectively ignored. Without these events, tandem duplications in $D[g_i]$ reduces to the single gene case, and by Theorem 1, $\{u_1, \ldots, u_k\}$ is tandem duplication eligible. □

Theorem 3. *Suppose nodes u and v in domain tree D are mapped to gene g and annotated by genes $g_1, \ldots, g_k$, and no losses occur in their subtrees D_u and D_v. If u, v are marked as duplication nodes by the reconciliation framework and are pairwise eligible in $D[g_i]$ for any g_i, then u_1, u_2 are pairwise eligible in $D[g_i]$ for all g_i.*

Proof. First note that the children of u (and v) must also be mapped to g. Otherwise, either a co-duplication event would be lower cost or there would be at least one loss between u (v) and one of its children. This means that u, v, and their children u_1, u_2, v_1, and v_2 are present in $D[g_i]\forall i$. Position values for these nodes will be the same across all $D[g_i]$, so if u, v are pairwise eligible in $D[g_i]$ for any g_i, then u_1, u_2 are pairwise eligible in $D[g_i]$ for all g_i. □

C Simulation Details

Our simulation consists of three components, a gene tree generator, a domain tree generator, and a sequence simulator. For the purpose of this work, we only use the first two components. Following the approach of other phylogenetic simulations [3], we generate gene trees and use these topologies to generate domain trees consistent with those gene level events. Our simulation adds the ability to have tandem duplications of multiple consecutive domains.

C.1 Gene Tree Generation

The inputs to our gene tree generation tool are the height h of the gene tree and the number of leaves n desired in the output topology. We use the createRandomTopology function from the Pyvolve [4] package to create a tree topology with n leaves. Branch lengths are assigned to branch in the gene tree such that the sum of distances on any root to leaf path is equal to h. Branch lengths are assigned top down, starting with the outgoing edges of the root, and ending with edges connected to the leaves of G. In the following, let $D(u, v)$ be the sum of branch lengths of all branches on a path from node u to v, while $d_T(u, v)$ (as defined in the main paper) is the topological distance, or number of nodes, on the path from u to v. Let r be the root node of G. For any internal node u, the remaining branch length to be assigned to branches on a path from u to any of its leaf descendants is $h - D(u, r)$. Let u_1 be a child of u, and v^* be the child of u_1 that minimizes $D(u_1, v^*)$. If u_1 is a leaf node, then $u_1 = v^*$. We assign branch (u, u_1) a length of $h - D(u, r)/(1 + D(u_1, v^*))$. After this process, we relax the branch lengths. For each branch we take the length l assigned by the process above and replace it with a value drawn from the normal distribution with mean l and standard deviation $l/10$.

C.2 Domain Tree Generation

Domain trees are generated with respect to a guest tree topology. We simulate duplication and loss events using a modified birth death process [5]. A birth death process starts with a single progenitor node, and domain duplications and losses ("births" and "deaths") occur with rates λ and μ respectively. Along any branch (u, v) with length l of the gene tree, evolutionary events occur to the domains belonging to node v. Distances between evolutionary events are drawn from an exponential distribution with mean $\lambda + \mu$. Let d be the "remaining distance" left on branch (u, v). Initially, d is set to l. A distance d_i is drawn from the exponential distribution, and if $d_i \leq d$, then an evolutionary event occurs and d is set to $d - d_i$. This process is repeated until at some step $d_i > d$. At this point, all domains present in gene v undergo a co-duplication event, resulting in a bifurcation at each of these domain nodes. The children of these domains are the starting domains initially present in the children of v, and the process repeats until the domain tree covers all genes in the gene tree.

Loss events affected single domains, while duplications could be tandem duplications of multiple consecutive duplications. When a loss event occurred, an existing domain was chosen at random and removed from the domain tree. The parent of this node was also removed to ensure the domain tree remained a full binary tree. When a duplication event occurred, we first chose the size of the tandem duplication such that the probability of a duplication of size k occurring was $\frac{1}{2^k}$. This allows for a variety of duplication sizes, while making shorter duplications more likely than longer ones. If a size k was drawn but the total number of duplications was less than k, then all domains on the gene duplicated simultaneously. Through this process, we keep track of the relative position of domains as follows:

1. The initial domain at the root of the gene tree has position 0

2. When the domain present in gene g at position i is lost, the positions of all domains in g with position greater than i are decremented by 1

3. If a tandem duplication of size k occurs in gene g affecting domains with positions $i, i+1, ..., i+k-1$, then one child of a duplicated domain with position j is assigned position j, while the other is assigned position $j+k$. For any domain that had a position $l > i+k-1$ prior to the duplication, we assign it a position of $l+k$.

4. For any domain with position i involved in a co-duplication event, we assign both of its children the position i

These rules are consistent with the tandem duplication assumptions laid out in Section 2.3 of the main paper.

C.3 Parameters Used in our Simulations

To test our methods, we generated 50 gene and domain tree pairs each at event distances of 0.01, 0.025, .05, .075, and 0.1. Our gene trees always had 8 leaves and a mean tree height of 0.3. Thus, the largest event distances resulted in very few domain level events, while the shortest ones frequently resulted in cascades of tandem duplications. For each event distance, we adjusted λ and μ based on the number of domains currently existing at the time of the event. We set these such that the probability of a duplication was higher when there were few domains, and the probabililty of a loss was higher once there were many (See Figure S6). This was done so that cascades of domains were more likely early one, followed by many losses. Due to the way position inference works, this represents the hardest scenario for our methods.

Figure S7 shows the sizes of the domain trees generated by our birth-death process at each event distance. At an event distance of 0.1, the average size of the domain tree is typically under 50 nodes, indicating that most genes contain no more than 2-3 domains total. Losses are rare at these event distances, so we see that very few duplications occur at this event distance. At larger event distances, we see much larger domain trees, indicating a much larger number of domains per gene. Here we expect many tandem duplication events across the tree, and several losses once the number of domains per gene crosses 10. Figure S8 looks specifically at the number of domains mapped to leaf genes. These represent the genes we see today, after evolutionary events have occurred across the gene tree. As we use only full binary trees, the number of leaf domains (representing contemporary domains in currently existing genes) per gene is at least half the total number of domains mapped to that gene. Again, we see that with a high event distance, the number of domains per leaf gene

Species	Sequence Accession Number	Number of Domains
Entamoeba Hystolytica	XP_657265.1	5
Trichoplax Adhaerens	XP_002118042.1	36
Nematostella Vectensis	XP_032219787.1	28
Hydra Vulgaris	XP_012557721.1	26
Drosophila Melanogaster	NP_001262657.2	22
Hirudo Medicinalis	AAR36862.1	35
Branchiostoma Floridae	XP_035677732.1	24
Ciona Intestinalis	XP_026692732.1	20
Danio Rerio	XP_009295045.1	23
Gallus Gallus	NP_989905.1	24
Taeniopygia Guttata	XP_012428163.2	24
Ornithorhynchus Anatinus	XP_028923661.1	24
Bos Taurus	NP_001193443.1	24
Mus Musculus	XP_006527974.1	24
Macaca Mulatta	XP_001091073.2	24
Pongo Abelii	XP_024096896.1	24
Pan Troglodytes	XP_016803150.1	24
Homo sapiens	sp|P21333|FLNA_HUMAN	24

Table 1: Table showing all 18 species used in our analysis, along with the sequence used and the number of filamin domains present in each sequence.

is typically only 1 or 2, while at the highest event distances over 20 domains can exist on a single gene.

D Filamin Dataset

We reanalyzed a filamin dataset from [2] to identify tandem duplications. 18 sequences were taken from 18 species, shown in Table 1. A total of 435 domains were found across these sequences. A domain tree was built from these sequences using RAxML [6] with the PROTGAMMAJTT amino acid substitution model, and reconciled against the gene tree given in [2]. As we have exactly one sequence from each species, the gene tree they give is identical to the species tree built from these species. This is shown in Figure S5.

References

[1] Chaitanya Aluru and Mona Singh. Identifying evolutionary origins of repeat domains in protein families. In *Proceedings of the 11th ACM International Conference on Bioinformatics, Computational Biology and Health Informatics*, pages 1–11, 2020.

[2] Sara Light, Rauan Sagit, Sujay S Ithychanda, Jun Qin, and Arne Elofsson. The evolution of filamin–a protein domain repeat perspective. *Journal of structural biology*, 179(3):289–298, 2012.

[3] Joel Sjöstrand, Lars Arvestad, Jens Lagergren, and Bengt Sennblad. Genphylodata: realistic simulation of gene family evolution. *BMC bioinformatics*, 14(1):1–5, 2013.

[4] Stephanie J Spielman and Claus O Wilke. Pyvolve: a flexible python module for simulating sequences along phylogenies. *PloS one*, 10(9):e0139047, 2015.

[5] Tanja Stadler. Simulating trees with a fixed number of extant species. *Systematic biology*, 60(5):676–684, 2011.

[6] Alexandros Stamatakis. Raxml version 8: a tool for phylogenetic analysis and post-analysis of large phylogenies. *Bioinformatics*, 30(9):1312–1313, 2014.

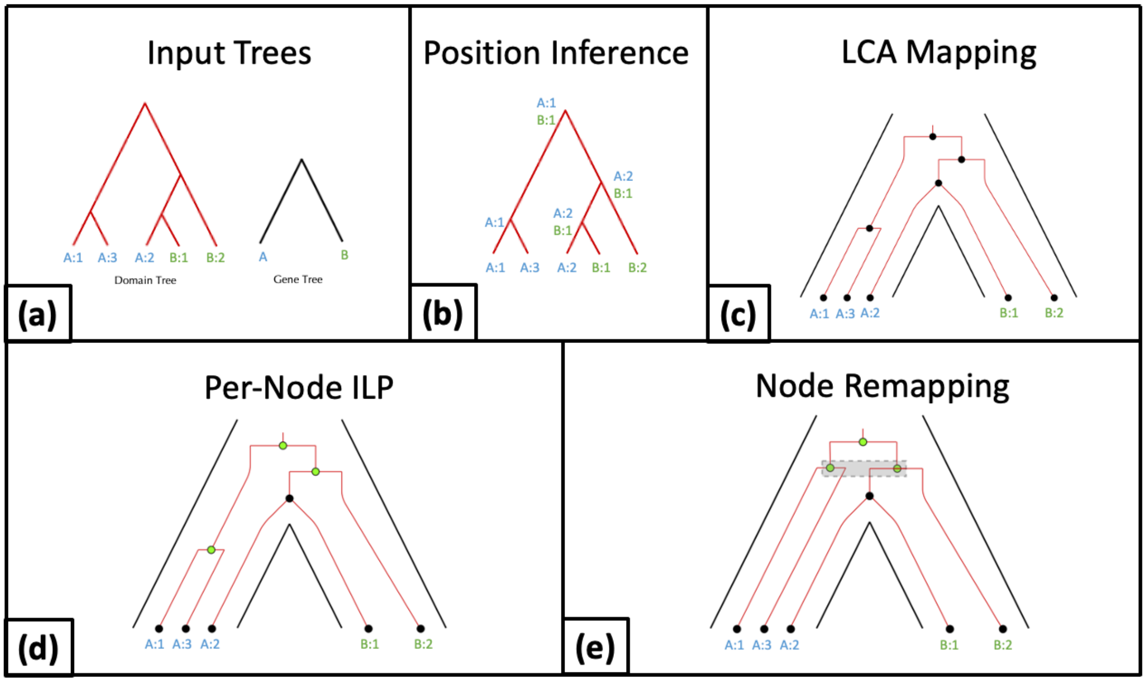

Figure S2: Full pipeline. **(a)** We take as input a domain tree and gene tree, represented as the red and black trees respectively. Leaf domains must be mapped to the genes they came from, and be annotated with their relative position on the gene with respect to other domains. In the figure, domains labelled X:Y are found in gene X with relative position Y. **(b)** We infer the position of each internal node in the domain tree based on the leaves in its subtree. For internal nodes whose subtrees contain leaf domains from multiple genes, we assign one position for each gene. Pairwise tandem duplication eligibility between any pair of internal nodes is then determined using the rules in Section 3 of the main paper. **(c)** In the first step of our heuristic, we create an initial mapping of internal domains to genes using a Lowest Common Ancestor (LCA) mapping. Although it is not guaranteed to be optimal for the TDL Reconciliation problem, in practice we expect very few nodes to be incorrectly mapped in this step. **(d)** For each gene node, a reduced ILP is run on the domain subtree(s) mapping to that node. If the mapping is correct, then this ILP will correctly identify a minimum cost solution to the TDL Reconciliation problem, including all tandem duplications and losses that occurred in that gene. Here, duplications are marked in green, while speciations/leaves are marked in black. **(e)** In the last step, we take the mapping previously found in step (c) and look for opportunities to remap tandem duplication sets to ancestral genes when doing so reduces overall cost. This can occur when two duplications can be merged into a single duplication, as in the figure.

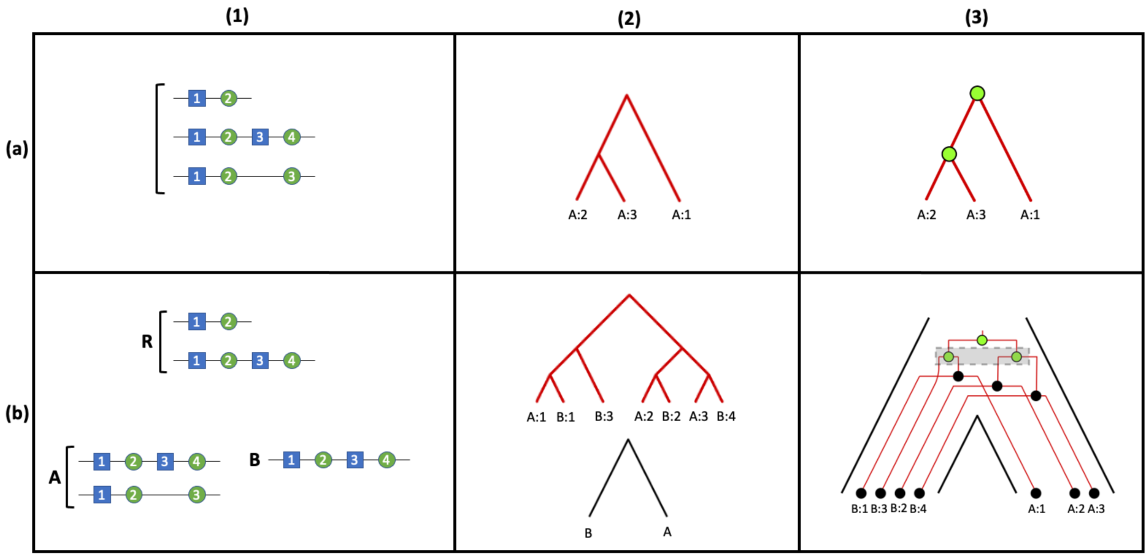

Figure S3: Here we show an example of how a loss can result in the incorrect inference of tandem duplications. In row **(a)**, we show an example of losses in domain evolution within a single gene. The actual evolutionary events are shown in **(a1)**. A single gene with two distinct domain instances (shown as the blue square and green circle) undergoes a tandem duplication event in which both domains are duplicated in tandem. Afterwards, the domain at position 3 is lost. **(a2)** shows the corresponding domain tree, and **(a3)** shows the duplication events inferred as green nodes. In this case, due to the loss of the second blue square domain, we no longer have the information required to identify the tandem duplication event, instead inferring that the ancestor of the domains in position 2 and position 3 underwent a single domain duplication. Row **(b)** shows how information from multiple genes can add robustness against these types of losses to our method. **(b1)** Here, a gene R initially contains two domains, which duplicate in tandem to result in 4 domains, two of each type. Through a speciation event, two copies of gene R are created, A and B. Afterwards, A independently loses domain 3, while B retains all four domains. **(b2)** shows the corresponding domain and gene trees in red and black respectively. Based on these trees, **(b3)** shows the reconciliation as well as the inferred duplication events (in green) and speciation events (in black). The gray box shows the inferred tandem duplication. In this case, because gene B retains the domains from the tandem duplication event, we are able to capture it even though gene A has a loss. Note that if the loss had occurred in gene R (and both genes A and B had inherited only 3 domains), then we would not be able to infer the existence of a tandem duplication event.

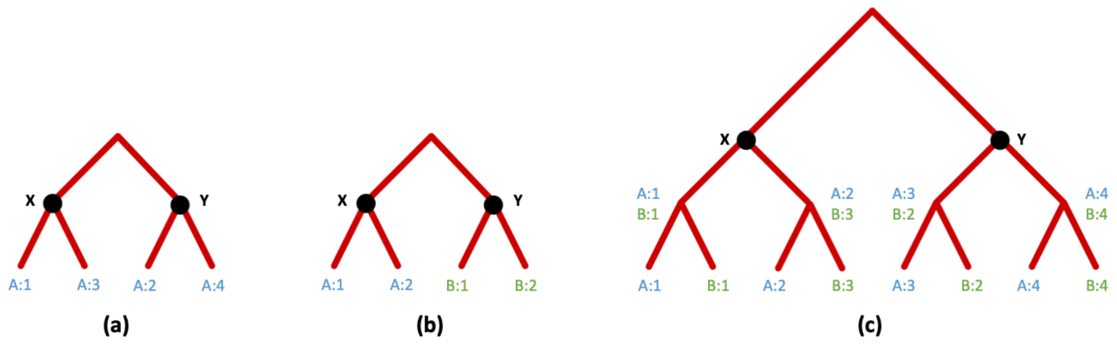

Figure S4: Examples of pairwise eligibility computation on internal domain nodes with 0, 1, or multiple annotations. As in previous examples, each leaf domain is labelled by the gene it occurs in and its relative position on the sequence, separated by a colon. We also provide per-gene annotations for other nodes in the tree when those positions are necessary to compute pairwise tandem duplication eligibility. In all cases, we decide whether nodes X and Y (represented as the black dots in each figure) are pairwise eligible or not. At this stage in the pipeline, we do not know which gene an internal domain will be mapped to, so X and Y are names that do not correspond to a particular gene. **(a)** In this case, nodes X and Y are both annotated by a single gene, A. We see that the positions of the leaves of X and Y are interleaved, and therefore X and Y are tandem duplication eligible. **(b)** In this example, nodes X and Y share no leaf genes in common. Therefore, we have no way to determine whether they are tandem duplication eligible, so we assume that they are. **(c)** In this example, X and Y are annotated by genes A and B. According to gene A, X and Y are not pairwise eligible. On the other hand, they are pairwise eligible according to gene B. This could be due to losses in the domain tree, errors in tree reconstruction, or events such as domain swaps which are not considered by our model. In this case, we are permissive and label X and Y as pairwise eligible because they are according to at least one gene annotation.

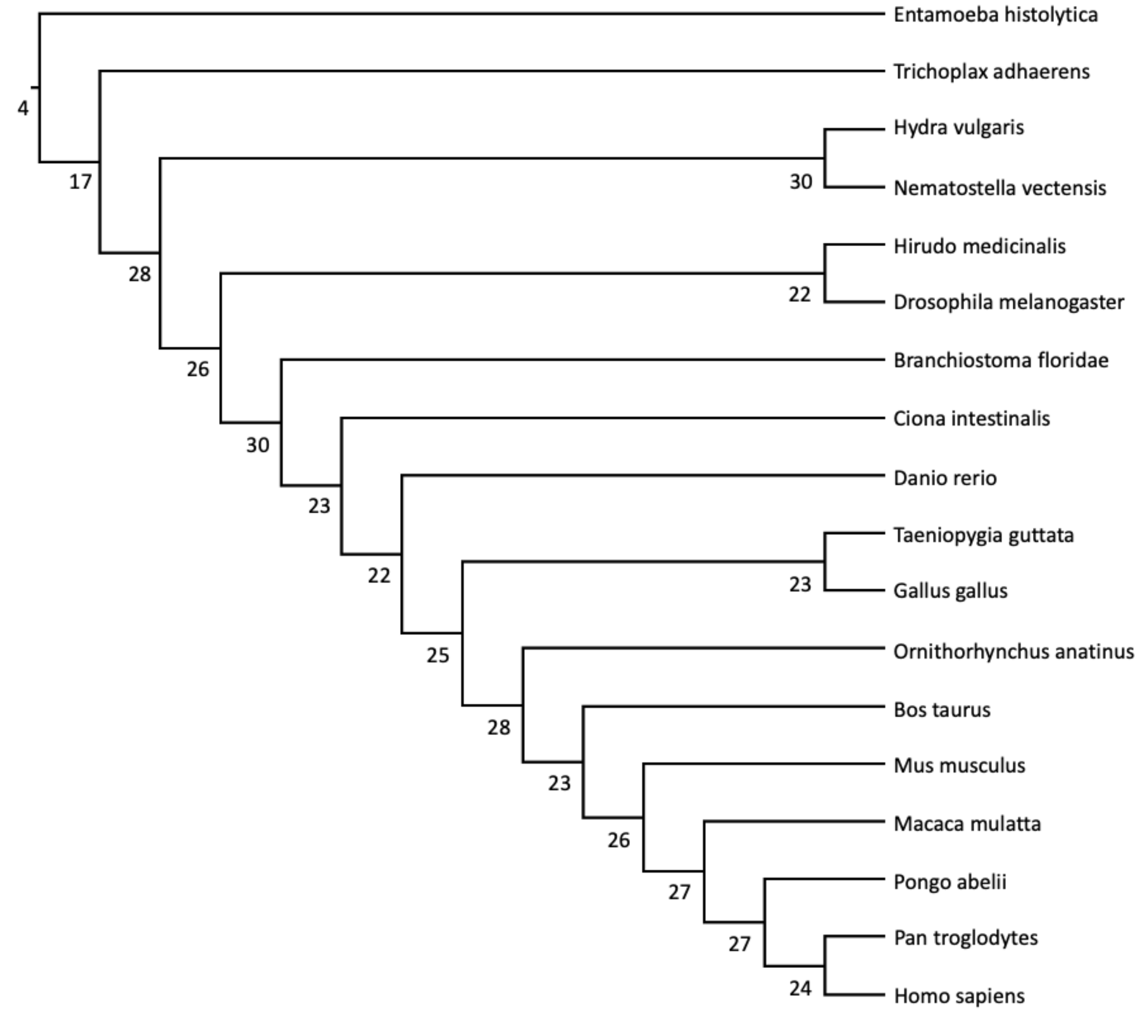

Figure S5: Gene tree, consisting of one gene each from 18 species, that is used for reconciliation in the Filamin domain example. The number at each internal node represents the number of Filamin A domains inferred to be in that ancestral sequence.

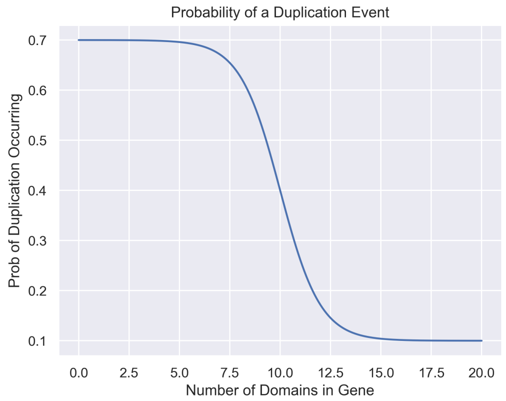

Figure S6: Duplication/Loss ratio function used for our duplication and loss probabilities. The probability of a duplication (of any size) occurring in a gene with k domains is shown. With a low number of domains, the probability of a duplication is 0.7. With a high number of domains, the probability of a duplication drops to 0.1. This encourages a series of tandem duplications early on, followed by mostly single domain losses once several duplication events have taken place.

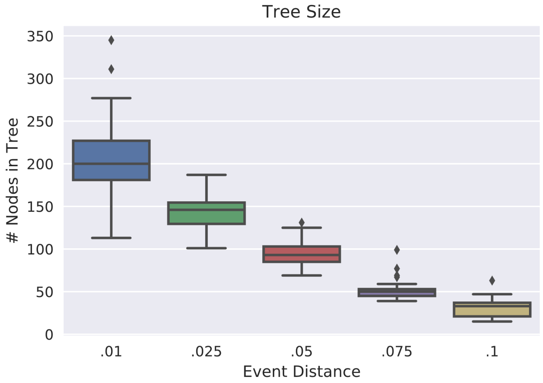

Figure S7: Boxplots of domain tree sizes. For each event distance, the corresponding boxplot shows the number of nodes in the domain tree for each example generated. A total of 50 gene/domain tree pairs were generated at each event distance. The size of the domain tree grows roughly linearly with decreasing event distance.

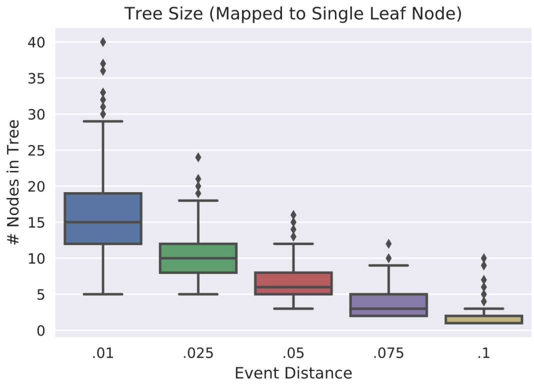

Figure S8: Boxplots of the number of domains in the domain subtrees mapped to leaf gene nodes. For each gene/domain tree example at a given event distance, the number of domain nodes per leaf gene is counted. 50 examples are present at each distance, and each gene tree has exactly 8 leaves, so the sizes of domain subtrees from 400 genes are shown at each distance. The total number of domains is typically between 1-3 at the highest event distance, while as many as 40 domains may be mapped to a single gene (indicating 20 coexisting domains) at the lowest event distance.
